# *Opuntia ficus-indica* as an Alternative Source of Mucilage in Low-Fat Ice Cream

**DOI:** 10.17113/ftb.63.03.25.8841

**Published:** 2025-09-29

**Authors:** Eduarda França Ferreira Souza, Amanda Kelly Cristiano Mafra, Raquel Guidetti Vendruscolo, Marcio Schmiele, Larissa de Oliveira Ferreira-Rocha

**Affiliations:** Institute of Science and Technology, Federal University of Jequitinhonha and Mucuri Valleys, MGT-367 Highway, Km 583, n. 5000, Diamantina, MG 39100-000, Brazil

**Keywords:** freeze-dried cactus pear cladode pulp, mucilage, low-fat ice cream, physicochemical properties, technological parameters

## Abstract

**Research background:**

Cactus pear (*Opuntia ficus-indica*) is an excellent source of polysaccharides and bioactive compounds with remarkable health benefits. The mucilage of the cactus pear, which consists mainly of water and complex carbohydrates, has properties similar to gum due to its unique physiological properties. Recently, plant-derived mucilage has gained significant attention in the dairy industry for its potential as a natural thickening and colloidal stabilizing agent.

**Experimental approach:**

This study investigates the use of freeze-dried cactus pear cladode pulp from *Opuntia ficus-indica* L. Miller as a source of mucilage and its interaction with a commercial stabilizer on the physical properties of low-fat cocoa ice cream (3.0 % fat). The study evaluates the influence of cactus pear cladode pulp on the physicochemical properties and technological parameters of the ice cream. Ice cream samples containing 1.0, 1.5 and 2.0 % cactus pear cladode pulp were compared with a control sample (without cactus pear cladode pulp).

**Results and conclusions:**

The results show that cactus pear cladode pulp is rich in fiber and phenolic compounds and has significant technological potential due to its water absorption capacity (WAC), water solubility index (WSI) and oil absorption capacity (OAC). The addition of cactus pear cladode pulp lowered the pH of the ice cream, improved its darkness and yellowness, increased the overrun and delayed the melting process. These results suggest that cactus pear cladode pulp works synergistically with the commercial stabilizer, highlighting its potential as a natural fat substitute and stabilizer for low-fat ice cream formulations.

**Novelty and scientific contribution:**

This study represents pioneering research into the use of freeze-dried *Opuntia ficus-indica* cladode pulp in the production of ice cream. The results offer valuable insights for the ice cream industry and provide a natural alternative for stabilizers and fat substitutes.

## INTRODUCTION

*Opuntia ficus-indica*, a member of the Cactaceae family, is mainly found in arid regions. The sustainable and mindful use of this plant can significantly contribute to the global goals of reducing poverty and hunger, while promoting sustainability and innovation in the food industry. The primary substance produced by *O. ficus-indica* is mucilage, a compound consisting mainly of water and polysaccharides. This mucilage plays a crucial role in the plant's adaptation mechanisms, as its cryostabilizing properties help to prevent dehydration or freezing. The mucilage extracted from the cladodes of the cactus pear is water-soluble and forms highly viscous colloidal solutions (*1*).

Structurally, the mucilage is a complex polymeric polysaccharide, consisting mainly of highly branched carbohydrate structures. These structures consist of monomeric units such as l-arabinose, d-xylose, d-galactose, l-rhamnose and galacturonic acid. In addition to these carbohydrates, mucilage may also contain glycoproteins and various bioactive components, including tannins, alkaloids and steroids. After hydrolysis, mucilage produces a heterogeneous mixture of monosaccharides that is commonly classified as gum-like due to its similar physicochemical properties (*2*).

Vegetable mucilage has gained considerable attention in the development of dairy products due to its effectiveness as a natural thickener and colloidal stabilizer (*3*). It has been extensively studied in yogurt production as it improves texture and minimizes whey separation during storage (*4*). Additionally, mucilage in products like fermented milk and cheese offers other benefits, including effective fat reduction (*5*–*7*), the potential to produce prebiotic foods (*5*, *6*, *8*, *9*) and support in the development of probiotic products (*6*, *8*).

Recent studies have investigated the use of vegetable mucilage in ice cream to replace or reduce the reliance on commercial and expensive additives. Mucilage serves as an effective cryoprotectant that compensates for the lack of various commercial stabilizers, while providing desirable technological and sensory properties (*10*–*13*). Since it is difficult to achieve all the desired properties of ice cream with a single stabilizer, the combination of two or three hydrocolloids in the mixture can produce synergistic effects. The amount and type of stabilizer needed depend on factors such as the type and strength of the stabilizer, the total solids and fat content of the mixture and other relevant factors (*14*).

There are few studies in the literature on the use of cactus pear mucilage as a fat substitute in low-fat ice creams. Therefore, the aim of this study is to investigate the use of freeze-dried cactus pear cladode pulp from *Opuntia ficus-indica* L. Miller as a source of mucilage and its interaction with a commercial stabilizer on the physical properties of ice cream. Additionally, the study aims to characterize the freeze-dried cactus pear clalode pulp by evaluating its physicochemical properties and technological parameters.

## MATERIALS AND METHODS

### Raw material and obtaining freeze-dried cactus pear cladode pulp

The project is registered with the Ministry of the Environment, a Brazilian organization that includes the National System for Management of Genetic Heritage and Associated Traditional Knowledge (protocol number: AF2C488). The cactus primary cladodes were collected in the municipality of Couto de Magalhães de Minas, in the state of Minas Gerais, located at an altitude of 740 meters and geographical coordinates of 18°04'16" south and 43°28'31" west.

The primary cladodes of the cactus pears were first cleaned with potable water to remove surface dirt, sanitized in a 200 mg/L sodium hypochlorite solution for 15 min, rinsed with potable water and then frozen until use.

**Fig. S1:**
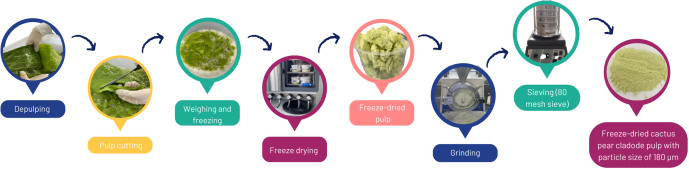
Process for obtaining freeze-dried cactus pear cladode pulp

Cactus pear cladode pulp was prepared as shown in Fig. S1. After thawing the cladodes overnight, the peel was removed to extract the pulp, which was cut into approx. 1.5 cm×1.5 cm pieces. These pieces were divided into 100-gram portions and freeze-dried at the MULTIFAR/PRPPG Center using a freeze dryer (FreeZone; Labconco®, Kansas, MO, USA) with the following parameters: temperature -50 °C and pressure 50 Pa.

The freeze-dried cactus pear cladode pulp was ground using a Willye-type macro mill (TE-650; Tecnal, Piracicaba, Brazil) and then sieved with an 80-mesh sieve using a sieve shaker (Bertel, Bertel, Caieiras, Brazil) to obtain a fine powder with a standard particle size of 180 μm. The ground and sieved cactus pear cladode pulp was packaged in polypropylene bags and stored in a refrigerator until use.

### Characterization of freeze-dried cactus pear cladode pulp

Moisture mass fraction was determined according to AOAC method 934.06 (*15*). Ash mass fraction was determined by burning the weighed mass of a sample in a muffle furnace according to AOAC 923.03 (*16*). Total dietary fiber (TDF), insoluble dietary fiber (IDF) and soluble dietary fiber (SDF) were analyzed using the AOAC method 991.43 (*17*). Protein mass fraction was determined with a CHNS/O elemental analyzer (TruSpec Micro; LECO, St. Joseph, MI, USA). Lipid content was analyzed using the Bligh and Dyer method (*18*). Total carbohydrate mass fraction was calculated as the difference between 100 % and the sum of the mass fractions (in %) of lipids, proteins and ash on a dry mass basis.

The pH was measured according to the electrometric method with a digital pH meter (mPA–210; MS Tecnopon, Piracicaba, Brazil) with a 1:10 sample dilution in distilled water (*19*). Water activity (*a*_w_) was determined at 25 °C using a water activity instrument (4TE Duo; AquaLab, Pullman, WA, USA).

Macrominerals (Ca, Mg, K and P) were analyzed with an atomic absorption spectrometer (SpectrAA 50B; Varian, Mulgrave, Australia).

The fatty acid profile was determined in two stages: extraction and chromatographic determination. The lipid fraction was extracted from water-soluble extracts using a mixture of methanol (Sciavicco, Belo Horizonte, Brazil), chloroform (Dinâmica, Química Contemporânea, Indaiatuba, Brazil) and water, according to the method described by Bligh and Dyer (*18*). Derivatization was then carried out according to the method described by Hartman and Lago (*20*). A volume of 1 mL of 0.4 M methanolic potassium hydroxide (Êxodo Científica, Sumaré, Brazil) solution was added to the lipid fraction and kept in a water bath (SL-150; Solab, Piracicaba, Brazil) at 100 °C for 10 min. The tubes were then cooled and 3 mL of 1 M methanolic sulfuric acid solution were added, followed by incubation at 100 °C for another 10 min. After cooling, 2 mL of hexane (Êxodo Científica) were added and the tubes were homogenized in a vortex (NA 3600; Norte Científica, Araraquara, Brazil) for 10 s. The upper layer containing the fatty acid methyl esters (FAMEs) dissolved in hexane was then collected for chromatographic analysis. The fatty acid profile was analyzed using gas chromatography with a flame ionization detector (GC-FID) (7820A; Agilent Technologies, Santa Clara, CA, USA). A 1 µL sample was injected in split mode with a 40:1 ratio at an injector temperature of 240 °C. Hydrogen was used as the carrier gas at a constant pressure of 103.4 kPa. Fatty acid methyl esters (FAMEs) were separated on a DB-23 capillary column (60 m×0.25 mm×0.25 µm; Agilent Technologies) under the following temperature program: an initial hold at 50 °C for 1 min, an increase to 175 °C at 25 °C/min, followed by an increase to 230 °C at 2 °C/min, with a final isothermal hold for 6 min. The detector temperature was set at 240 °C. FAMEs were identified by comparing retention times with those of the FAME Mix 37 standard (P/N 47885; Sigma-Aldrich, Merck, St. Louis, MO, USA) (*21*). Results were expressed as the percentage of the total chromatogram area, which incorporated FID correction factors and accounted for the conversion of esters to acids (*22*).

Water absorption capacity (WAC) and water solubility index (WSI) were determined following the method described by Schmiele *et al*. (*23*). Oil absorption capacity (OAC) was measured using the method described by Benítez *et al*. (*24*).

The color of the freeze-dried cactus pear cladode pulp was evaluated using instrumental colorimetry according to the CIE Lab* system, with illuminant D65, a 10° viewing angle and calibration in SCI (specular component included) mode using a spectrophotometer (CM-5; Konica Minolta, Chiyoda, Japan). The CIE parameters evaluated were: L* value (100=white; 0=black), a* (+ red, - green) and b* (+ yellow, - blue).

The total phenolic content (TPC) was determined using a modified method described by Nascimento *et al.* (*25*), which ensures improved accuracy and reproducibility. The TPC were extracted with a water/acetone ratio of 52:48 (Isofar, Duque de Caxias, Brazil) in six cycles, with the supernatant adjusted to a final volume of 10 mL. For the color reaction, 100 μL of the extracting solution containing phenolics, 250 μL of the 0.2 M Folin-Ciocalteu reagent (Êxodo Científica), 3 mL of distilled water and 1 mL of 15 % Na_2_CO_3_ solution were used. The reaction was developed in the dark for 30 min. A standard curve was prepared with gallic acid. Absorbance was measured at 750 nm with a spectrophotometer (UV-M5; Bel Photonics, Monza, Italy). Readings were taken in six replicates and results were expressed in milligrams of gallic acid equivalents per 100 grams of sample (dry mass basis). A blank containing the extraction solvent was used to zero the instrument.

### Raw materials and ingredients for ice cream preparation

The raw materials and ingredients for the preparation of the ice creams were: water; skimmed milk powder containing 50 % carbohydrate, 34.5 % protein and 0 % fat (Itambé®, Belo Horizonte, Brazil); 35 % fat cream (Itambé®, Belo Horizonte, Brazil); sucrose (Delta®, Delta, Brazil); inverted sugar (Ingredientes Online, São Paulo, Brazil); dextrose (Ingredientes Online, São Paulo, Brazil); stabilizer (Super Liga Neutra; Selecta®, Jaraguá do Sul, Brazil); emulsifier (Emustab, Selecta®, Jaraguá do Sul); freeze-dried cactus pear cladode pulp and cocoa powder (Sicao®, Extrema, Brazil).

### Ice cream formulation and preparation

The ice cream mixtures were prepared with a balanced syrup with a final fat content of 3.0 % (low-fat) based on the total syrup volume (Table 1). The amount of freeze-dried cactus pear cladode pulp varied from 0.0 (control) to 2.0 %.

**Table 1:** Composition of chocolate ice cream formulations

Ingredient	*w*(ingredient)/%			
	C	F1	F2	F3
Water	53.28	53.28	53.28	53.28
Skimmed milk powder	13.96	13.96	13.96	13.96
Cream*	7.07	7.07	7.07	7.07
Sucrose	6.43	6.43	6.43	6.43
Inverted sugar	7.35	7.35	7.35	7.35
Dextrose	5.05	5.05	5.05	5.05
Stabilizer	0.94	0.94	0.94	0.94
Emulsifier	0.94	0.94	0.94	0.94
Cocoa powder	2.98	2.98	2.98	2.98
Freeze-dried cactus pulp	0	1.00	1.50	2.00

*Cream contains 35 % fat

The production of the ice cream began with the dissolution of the ingredients (Table 1) in water at 40 °C, with the exception of the cocoa powder. The mixture was blended and homogenized in an industrial blender (model LQI-06; Vitalex, Catanduva, Brazil). It was then pasteurized at 70 °C for 30 min, cooled and left to mature. After maturation, the cocoa powder was added to the mixture using the same industrial blender. The flavored mixture was then churned and frozen in a batch freezer (V-5; FortFrio, Betim, Brazil). Finally, the ice cream was hardened in a freezer at -18 °C.

### Overrun, meltdown rate and color

The air incorporation rate, or overrun (%), was calculated using the following equation:


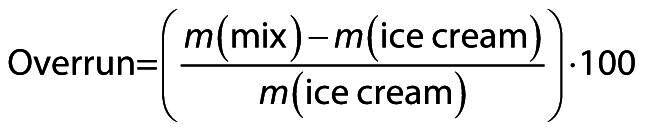
 /1/

The meltdown rate and the first dripping time of the ice cream samples were measured using methods adapted from previous studies (*11*, *26*). To determine the melting rate, samples (30 g) were stored at -12 °C (service temperature) for 24 h. Each sample was then placed in an incubation chamber at (25±1) °C, on a sieve with a mesh size of 1.25 cm, positioned over a pre-weighed beaker. The melted material passing through the sieve was collected and weighed at 5-minute intervals for approx. 30 min using an electronic digital balance (±0.01 g) (S2202H; Bel, Piracicaba, Brazil). The results were analyzed by plotting the melted ice cream mass against time. Linear equations were generated to determine the melting rate (*27*). The moment the first drop was observed was recorded as the initial dripping time (*28*).

The color of the ice cream was evaluated in the same way as described for color determination of the freeze-dried cactus pear cladode pulp. Total color difference was determined according to the following equation:


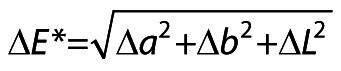
 /2/

The experiment was conducted in triplicate for all samples.

### Statistical analysis

The data were analyzed by Analysis of Variance followed by Tukey's test at the 5 % significance level, using Sisvar v. 5.8 software (*29*).

## RESULTS AND DISCUSSION

### Characterization of freeze-dried cactus pear cladode pulp

Table 2 summarizes the approximate composition of freeze-dried cactus pear cladode pulp. The freeze-dried cactus pear cladode pulp had a lower final moisture mass fraction than the maximum limit of 15 % for vegetable flour established by RDC Nº. 711, dated 1 July 2022 (*30*). This low moisture mass fraction ensures microbiological stability and makes the freeze-dried cactus pear cladode pulp suitable for preservation and storage.

**Table 2:** Characterization of freeze-dried cactus pear cladode pulp

Compound	*w*(compound)/%
Protein	8.17±0.00
Lipid	1.46±0.05
Ash	16.85±0.05
IDF	26.1±3.1
SDF	17.3±1.1
TDF	43.3±2.0
Carbohydrate	73.5±0.4
K	4.9±1.9
Ca	3.2±1.7
Mg	0.9±0.1
P	0.15±0.02
Moisture	8.5±0.3
Total solids	91.5±0.3
Other properties	
*w*(TSPC as GAE)/(mg/100 g)	1242±49
*a*_w_	0.37±0.00
pH	4.35±0.08

Results are expressed as mean value±standard error, *N*=3. IDF, SDF and TDF=insoluble, soluble and total dietary fiber, respectively, TSPC=total soluble phenolic compounds, GAE=gallic acid equivalent

The water activity (*a*_w_) of the freeze-dried cactus pear cladode pulp was 0.37 and the pH was 4.35. With an *a*_w_ below 0.6 and the observed pH value, the freeze-dried cactus pear cladode pulp is well-protected against the growth of deteriorating microorganisms (*31*). The low pH can be attributed to the presence of organic acids, including malic, citric and oxalic acid (*32*).

The moisture mass fraction and *a*_w_ of cactus pear mucilage vary among species, with differences influenced mainly by the variety and the harvesting season (dry *vs* rainy). Higher moisture mass fractions are associated with a shorter shelf life (*33*, *34*).

Proteins are essential for the formation of foam and emulsions, which function as surfactants at air-water (surface property) or oil-water (hydrodynamic property) interfaces. They form a highly viscoelastic film that can withstand mechanical stress and gravity (*35*). The protein mass fraction in freeze-dried cactus pear cladode pulp was 8.17 %. This result is consistent with Gebremariam *et al*. (*34*), who reported a protein mass fraction of approx. 8 % in the dry matter of cactus pears (*Opuntia ficus-indica*). In contrast, Du Toit *et al*. (*33*) observed a protein mass fraction between 3.28 and 3.64 % in powdered mucilage, with no significant variation during a 6-month harvesting period. Similarly, Du Toit *et al.* (*36*) reported protein mass fractions of 2.7 to 3.2 % in *O. ficus-indica*.

The lipid mass fraction of the freeze-dried cactus pear cladode pulp was 1.46 %. Yadav *et al*. (*37*) suggest that the lipid content in gums plays a significant role in reducing surface tension, thereby enhancing the stability of oil-in-water emulsions. A similar lipid mass fraction of 1.19 % was reported by Dick *et al*. (*38*) in powdered mucilage of *Opuntia monacantha*.

The ash mass fraction determined in this study was 16.8 %, which is higher than the 15.14 % reported by Dick *et al*. (*38*) in powdered mucilage from *Opuntia monacantha* cladodes. However, it is lower than the ash content reported in previous studies for *Opuntia ficus-indica* cladodes, where Malainine *et al*. (*39*) determined values up to 19.6 %.

The total phenolic content, expressed as gallic acid equivalents (GAE), in the freeze-dried cactus pear cladode pulp was 1242 mg/100 g. Polyphenols are known to be the main contributors to antioxidant activity. Numerous studies have focused on the identification of natural antioxidants in low-cost raw materials. For instance, apple pomace, with a phenolic content, expressed as GAE, of 1016 mg/100 g, is considered a significant source of natural polyphenols. Similarly, cactus pear cladodes can be considered a valuable and economical source of natural antioxidants (*40*).

The freeze-dried cactus pear cladode pulp has a significant mineral content measured on dry mass basis, especially potassium (4.9 %), calcium (3.2 %) and magnesium (0.9 %). These results are consistent with the mineral profile usually reported for the genus *Opuntia ficus-indica*, where potassium and calcium are typically found in higher amounts, followed by magnesium (*41*). The mineral composition of *Opuntia ficus-indica* is remarkable because the calcium contained in the mucilage is bioavailable and can be absorbed in the human gastrointestinal tract. This suggests that *Opuntia* mucilage could have new and important applications in the food industry (*42*).

The freeze-dried cactus pear cladode pulp contained a 43.3 % total dietary fiber (TDF), of which 26.1 % was insoluble fiber (IDF) and 17.3 % soluble fiber (SDF). This composition is consistent with the results of *Opuntia ficus-indica* f. *amyloceae* (spiny cladodes), which had a TDF content of 51.24 % and an SDF/IDF ratio of 1:3 (*40*). The higher mass fraction of IDF than of SF in freeze-dried cactus pear cladode pulp is consistent with these results and underlines the typical fiber distribution in *Opuntia* species.

The fatty acid profile of the freeze-dried cactus pear cladode pulp, shown in Table 3, shows a predominant composition of polyunsaturated fatty acids (PUFA) at 54.01 %, with linoleic acid (18:2n-6) being the most abundant. Among the saturated fatty acids (SFA), palmitic acid had the highest mass fraction of 26.9 %. Oleic acid (9.4 %) was the only monounsaturated fatty acid (MUFA) found. This profile is consistent with that of chia mucilage, which also contains nutritionally beneficial fatty acids, further highlighting the potential of freeze-dried cactus pear cladode pulp as a valuable nutritional ingredient (*13*).

**Table 3:** Fatty acid profile of freeze-dried cactus pear cladode pulp

Fatty acid	IUPAC nomenclature	Common nomenclature	*w*(fatty acid)/%
SFA			
C6:0	Hexanoic acid	Caproic acid	0.23±0.00
C8:0	Octanoic acid	Caprylic acid	0.30±0.01
C12:0	Dodecanoic acid	Lauric acid	0.34±0.01
C13:0	Tridecanoic acid	Tridecylic acid	0.50±0.01
C14:0	Tetradecanoic acid	Myristic acid	0.69±0.03
C16:0	Hexadecanoic acid	Palmitic acid	26.9±0.5
C17:0	Heptadecanoic acid	Margaric acid	1.29±0.03
C18:0	Octadecanoic acid	Stearic acid	2.54±0.01
C24:0	Tetracosanoic acid	Lignoceric acid	3.9±0.1
∑SFA			36.63
MUFA			
C18:1n9c	9-Octadecenoic acid (*cis*)	Oleic acid	9.4±0.2
∑MUFA			9.36
PUFA			
C18:2n6c	*cis*-9, *cis*-12-Octadecadienoic acid	Linoleic acid	41.6±0.6
C18:3n3	*cis*-9, *cis*-12, *cis*-15-Octadecatrienoic acid	Linolenic acid	12.4±0.2
∑PUFA			54.01
n6/n3			3.35

Results are expressed as mean value±standard error, *N*=3. SFA, MUFA and PUFA=saturated, monounsaturated and polyunsaturated fatty acids, respectively, n6/n3=ratio between omega-6 and omega-3 fatty acids

The physical properties of the freeze-dried pulp of *Opuntia ficus-indica* L. Miller are shown in Table 4. The hydration properties of the freeze-dried *Opuntia ficus-indica* L. Miller pulp were characterized by a water solubility index (WSI) of 42.4 % and measured on dry mass basis a water absorption capacity (WAC) of 5.6 g/g, with an oil absorption capacity (OAC) of 2.93 g/g. This high fiber mass fraction is responsible for the remarkable hydration properties. On the other hand, hydrophobic components, particularly the apolar radicals of proteinaceous amino acids, contribute to the oil absorption capacity. Additionally, dietary fiber can adsorb some oil on their surface, which further influences OAC values (*42*).

**Table 4:** Physical properties of freeze-dried cactus pear cladode pulp

Parameter	Result
WAC/(g/g)	5.6±0.1
WSI/%	42.4±8.3
OAC/(g/g)	2.93±0.02
L*	79.9±0.1
a*	-7.11±0.05
b*	27.1±0.3

Results are expressed as mean value±standard error, *N*=3. WAC=water absorption capacity, WSI=water solubility index, OAC=oil absorption capacity, WAC and OAC were determined on dry mass basis

The freeze-dried cactus pear cladode pulp had a high luminosity value (L*=79.9), a negative a* value (-7.11), indicating a green hue, and a positive b* value (27.1), suggesting a yellowish tint. This combination of values reflects a greenish-yellow color of the freeze-dried pulp, as described in Table 4. The observed color can be attributed to the presence of natural pigments such as chlorophylls and carotenoids, or tannic substances from the tegument of the cactus pear (*43*).

### Physical properties of ice cream

Table 5 shows the average results for total solids, pH, overrun and instrumental color parameters of the ice cream, as well as the visual representation of the ice cream colors. The total solids content showed no significant difference (p>0.05) between the formulations, indicating that the addition of freeze-dried cactus pear cladode pulp did not affect this parameter. The only variation among the formulations was in the amount of mucilage, which ranged from 0.0 to 2.0 % (Table 1).

**Table 5:** Evaluated parameters of physical properties of ice cream samples

Parameter	C	F1	F2	F3
*w*(total solid)/(g/100 g)	42.0±0.8	41.61±0.05	40.9±0.1	41.2±0.6
pH	(6.42±0.05)^a^	(6.16±0.01)^b^	(6.01±0.01)^c^	(5.94±0.00)^d^
Overrun/%	(19.83±0.00)^d^	(20.23±0.00)^c^	(26.79±0.00)^b^	(33.59 ±0.00)^a^
L*	(43.7±0.6)^a^	(39.9±1.0)^b^	(41.0±0.8)^ab^	(41.2±1.5)^ab^
a*	(11.0±0.3)^ab^	(10.0±0.5)^c^	(10.54±0.09)^bc^	(11.4±0.1)^a^
b*	(14.0 ±0.2)^d^	(15.1±0.3)^c^	(18.1±0.1)^b^	(20.1±0.3)^a^
ΔE*	0.00	4.09	4.94	6.60
Melting rate/(g/min)	0.7±0.1	0.70±0.07	0.6±0.2	0.64±0.04
*t*(first dripping)/min	(11.5±1.2)^c^	(10.2±0.8)^c^	(13.0±0.5)^ab^	(14.7±0.3)ª

Results are expressed as mean value±standard error, *N*=3. Δ*E*=total color difference. Different letters in the same row indicate significant differences between samples at the 5 % significance level, according to the Tukey’s test. C=control (without freeze-dried cactus pear cladode pulp), F1, F2 and F3=ice cream with 1.0, 1.5 and 2.0 % freeze-dried cactus pear cladode pulp, respectively

The pH values of the ice cream ranged from 5.94 (F3) to 6.42 (control sample), which is consistent with existing literature that reports pH values between 6 and 7 for ice creams with mucilage (*11*, *12*, *44*). The results show that the freeze-dried cactus pear cladode pulp significantly affected pH (p<0.05), with higher mass fractions of freeze-dried cactus pear cladode pulp leading to lower pH values. This effect is similar to the results with quince seed powder in ice cream, where acidic compounds contributed to lower pH values, despite the buffering effect of milk proteins (*45*).

The ice creams had intermediate brightness values (L*=39.9–43.7), positive a* values (10.0–11.4), indicating a reddish hue, and positive b* values (14.0–20.1), indicating a yellowish tint. This resulted in a brownish color of the ice creams, as shown in Table 5. The observed color is consistent with the use of cocoa in the formulation, which gives the product a characteristic brown hue.

There was a significant difference (p<0.05) in the color of the ice creams, with higher mass fractions of freeze-dried cactus pear cladode pulp leading to decreased L* values and increased b* values, indicating that the products became darker and more yellow. The freeze-dried cactus pear cladode pulp itself had a yellowish-green color, as detailed in Table 4. While the a* parameter of freeze-dried cactus pear cladode pulp did not notably affect the ice cream color—since the positive a* values were primarily due to the cocoa addition—there was a discernible color change compared to the control. According to Adekunte *et al*. (*46*), perceptible color differences can be classified as very distinct (Δ*E*>3), distinct (1.5<Δ*E*<3) and small differences (Δ*E*<1.5). All samples showed a very distinct color compared to the control sample. It can be observed that the higher the mass fraction of freeze-dried cactus pear cladode pulp, the greater the color difference, indicating that the cactus contributed to changing the color perception of the ice creams.

The incorporation of air into ice cream, known as overrun, is a critical physical property that affects the texture, softness and stability of the ice cream (*12*). In this study, increasing the mass fraction of freeze-dried cactus pear cladode pulp in the formulation led to a significant increase (p<0.05) in overrun, with values ranging from 19.83 to 33.59 %.

The overrun values in ice creams with chia seed mucilage ranged from 25 to 55 % (*13*), while those with quince seed powder ranged from 26.94 to 30.03 % (*45*). Ice cream with chia powder had overrun values between 18.82 and 40.21 % (*44*), and low-fat ice cream generally showed values between 11.64 and 34.68 % (*26*).

The increase in overrun due to the addition of freeze-dried *O. ficus-indica* pulp represents a potential advantage for the dairy industry. Increased overrun percentages contribute to improved texture and stability of ice cream by reducing the formation of ice crystals and improving product consistency during storage (*47*). This effect is partly due to the cryostabilizing properties of certain polysaccharides in cactus pear cladode pulp (*48*).

Effective resistance to melting and shape retention are essential quality characteristics for ice cream. Rapid melting can lead to a loss of structure before consumption, which has a negative impact on consumer satisfaction. Conversely, an excessively slow melting rate can be an indication of possible defects that point to problems with the formulation or processing of the ice cream (*14*).

Specifically, ice creams with higher mass fractions of freeze-dried cactus pear cladode pulp had melting times of 14.7 and 13.0 min for formulations F3 and F2, respectively, indicating greater resistance to the onset of melting (Table 5). In contrast, there was no significant difference (p<0.05) in the melting rate.

**Fig. 1:**
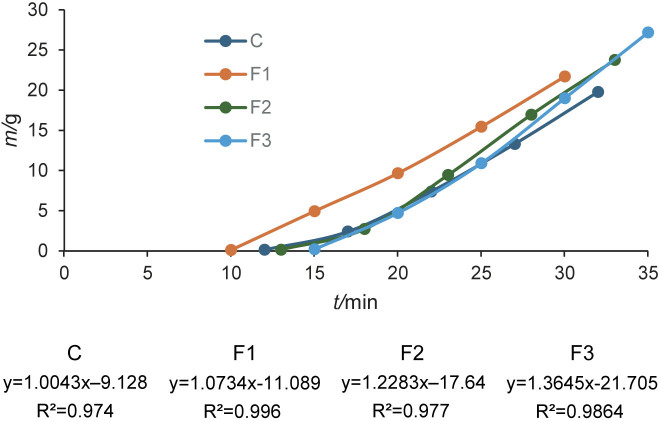
Mass loss of ice cream samples during melting: C=control ice cream, without the addition of freeze-dried cactus pear cladode pulp, F1, F2 and F3=ice cream with 1, 1.5 and 2.0 % freeze-dried cactus pear cladode pulp, respectively

Fig. 1 shows the melting properties of the different ice cream formulations, showing the behaviour of the ice cream regarding dripping time and melting rate. It was observed that the time to the first drip increased with higher mass fractions of freeze-dried cactus pear cladode pulp, demonstrating that the addition of cactus pear cladode pulp delayed the onset of melting. However, once melting began, the melting rate remained unchanged.

Similar results were observed in ice creams containing basil seed gum, guar gum and their mixtures, where these ingredients also contributed to improved melting resistance in low-fat ice creams (*23*).

A reduction in melting times was observed with the addition of quince seed powder, which has a high polysaccharide and protein contents (*48*). Similarly, chia seed mucilage powder reduced the melting rate in ice creams compared to samples without stabilizers, which is consistent with the results of studies using chia seed mucilage as a stabilizer (*11*). Previous studies indicate that high amounts of chia seed mucilage lead to increased melting resistance, attributed to the high viscosity of the ice cream mixture (*10*).

## CONCLUSIONS

The freeze-dried forage cactus cladode pulp has a high content of dietary fiber and phenolic compounds, and has a considerable technological potential due to its water absorption capacity (WAC), water solubility index (WSI) and oil absorption capacity (OAC). Ice cream with higher mass fractions of cactus pear cladode pulp had greater resistance to melting, characterized by longer melting onset times. These results suggest that the freeze-dried forage cactus cladode pulp is a promising fat substitute and stabilizer for low-fat ice creams, with the most effective results obtained at a mass fraction of 2.0 % freeze-dried cactus pear cladode pulp. Future research should focus on the extraction and application of pure mucilage in ice cream formulations, as well as rheological studies and sensory evaluations to further optimize and improve the results obtained.
